# Comprehensive biomedicine assessment of *Apteranthes tuberculata* extracts: Phytochemical analysis and multifaceted pharmacological evaluation in animal models

**DOI:** 10.1515/med-2024-1092

**Published:** 2025-06-03

**Authors:** Sajida Afzal, Siraj Khan, Muhammad Imam Ammarullah

**Affiliations:** Department of Biomedical Engineering (Medicine), Shenzhen University, Shenzhen, 518060, Guangdong, China; Shenzhen Hospital, Shenzhen, Guangdong, China; Qarshi Herb Research Center at Qarshi Industries (Pvt.) Ltd, Haripur, Hattar, Pakistan; Qarshi University, Lahore, 54000, Pakistan; Department of Mechanical Engineering, Faculty of Engineering, Universitas Diponegoro, Semarang, 50275, Central Java, Indonesia; Undip Biomechanics Engineering & Research Centre (UBM-ERC), Universitas Diponegoro, Semarang, 50275, Central Java, Indonesia; Bioengineering and Environmental Sustainability Research Centre, University of Liberia, Monrovia, 1000, Montserrado, Liberia

**Keywords:** *Apteranthes tuberculata*, extraction methods, phytochemical analysis, antioxidant activity, animal studies

## Abstract

**Background:**

This study comprehensively analyzes the pharmacological effects of ethanolic extract of *Apteranthes tuberculata* (EEAT) on various physiological parameters in mice.

**Objective:**

The research aimed to quantify flavonoid and phenol contents across different extraction methods, with a focus on the superior efficacy of the ethanolic extract.

**Methods:**

*In vitro* assays were conducted to assess the antioxidant activity of EEAT, revealing dose-dependent effects and significant inhibition percentages.

**Results:**

EEAT exhibited notable analgesic effects in the writhing response test, particularly at 200 mg/kg, indicating its potential as a natural analgesic. Additionally, its anti-inflammatory effects were complex and dose-dependent in a carrageenan-induced paw edema model. The extract also showed significant changes in body temperature regulation following Brewer’s yeast-induced fever, revealing a distinct pattern of initial hypothermia followed by gradual re-elevation. Furthermore, EEAT demonstrated regulatory effects on gastrointestinal motility, with higher doses enhancing intestinal transit in charcoal meal tests.

**Conclusion:**

This study highlights the pharmacological potential of EEAT as a natural therapeutic agent for antioxidant, analgesic, anti-inflammatory, and gastrointestinal regulation, warranting further investigation into its mechanisms of action and therapeutic applications.

## 1 Introduction

Medicinal plants have played an indispensable role in healthcare systems for centuries, serving as vital sources of bioactive compounds with significant therapeutic potential. Throughout history, natural products have formed the cornerstone of pharmacopoeias in traditional medicine practices worldwide, where plant-derived remedies have been employed to treat a wide range of ailments. In recent years, the exploration of medicinal plants has gained renewed interest, particularly with advancements in pharmacological research and biotechnology. These developments have enabled the identification, isolation, and synthesis of bioactive compounds from plants, shedding light on their pharmacological properties and therapeutic potential in modern medicine [1]. As the demand for novel and safer therapeutics continues to rise, medicinal plants remain a rich reservoir for discovering new drugs with diverse pharmacological profiles [2].

The resurgence of interest in medicinal plants has been especially notable in the field of phytochemistry, where the detailed analysis of plant constituents has uncovered a vast array of bioactive molecules. Phytochemicals such as flavonoids, phenolic acids, alkaloids, and terpenoids, commonly found in medicinal plants, have attracted significant attention for their antioxidant, anti-inflammatory, antimicrobial, and analgesic properties [3]. As research progresses, the role of these bioactive compounds in modulating biochemical pathways and addressing oxidative stress, inflammation, and microbial infections has become increasingly evident. Consequently, medicinal plants are seen as promising candidates for addressing global health challenges, especially as resistance to conventional drugs and synthetic agents becomes a growing concern.

Among the many plants under scientific investigation, *Apteranthes tuberculata*, a member of the Acanthaceae family, stands out for its rich phytochemical composition and purported medicinal benefits [4]. Historically utilized in traditional medicine, *A. tuberculata* has been reported to contain various bioactive compounds, including flavonoids, phenols, alkaloids, and terpenoids, each contributing to its therapeutic potential. These compounds have been linked to a range of pharmacological activities, such as antioxidant, anti-inflammatory, analgesic, antimicrobial, and antidiabetic effects [5]. While phytochemical studies have revealed the presence of these bioactive compounds, further research is needed to fully elucidate their pharmacological mechanisms, particularly through *in vivo* investigations.

Despite its long history of traditional use and promising phytochemical profile, comprehensive *in vivo* studies on *A. tuberculata* remain limited. The majority of studies to date have focused on *in vitro* analyses, which, although valuable for understanding the biochemical properties of plant extracts, do not provide a complete picture of their physiological effects in living organisms [6]. *In vivo* studies are crucial for evaluating the therapeutic efficacy and safety of bioactive compounds, as they account for complex biological interactions, metabolism, and toxicity [7]. Thus, a deeper understanding of the pharmacological potential of *A. tuberculata* requires well-designed *in vivo* experiments that assess its effects on various physiological parameters, including antioxidant activity, inflammation, pain modulation, and body temperature regulation.

This research article aims to fill this gap by conducting a comprehensive pharmacological evaluation of the ethanolic extract of *A. tuberculata* (EEAT) in animal models. The study will focus on assessing the extract’s effects on key physiological and pharmacological outcomes, including antioxidant capacity, analgesic effects, anti-inflammatory properties, and thermoregulation. By using *in vivo* models, the present study aims to provide critical insights into the therapeutic potential of *A. tuberculata* extracts, while phytochemical analyses will elucidate the specific bioactive compounds responsible for the observed effects. The findings of this research will not only contribute to the understanding of *A. tuberculata*’s bioactivity but also support its potential for drug development and modern therapeutic applications, thus advancing the role of medicinal plants in addressing contemporary medical challenges

## 2 Materials and methods

### 2.1 Plant material and extract preparation

The plant material of *A. tubercula*ta was collected from the Karak District in Khyber Pakhtunkhwa, Pakistan, during its natural growing season. Botanical authentication of the species was performed by an experienced taxonomist, and a voucher specimen was deposited in the herbarium for future reference. The freshly harvested plant material was thoroughly cleaned to remove soil and debris, followed by shade drying at ambient temperature to preserve its phytochemical integrity. Once dried, the plant material was finely ground using an electric grinder to obtain a homogeneous powder for extraction purposes.

For extract preparation, 100 g of the powdered plant material was subjected to maceration with different solvents, including methanol, ethanol, and distilled water, in accordance with well-established extraction protocols [8]. Each solvent was chosen to ensure a broad spectrum of phytochemicals was extracted based on their solubility profiles. The plant powder was soaked in each solvent separately at room temperature for 72 h with intermittent agitation to enhance solvent penetration and maximize extraction efficiency. After the extraction period, the mixtures were filtered through Whatman No. 1 filter paper to remove particulate matter. The filtrates were concentrated under reduced pressure using a rotary evaporator at 40°C to avoid thermal degradation of sensitive compounds. The resulting concentrated extracts were then lyophilized to obtain dry crude extracts, which were stored at −20°C for subsequent analyses.

### 2.2 Phytochemical composition analysis

The crude extracts were subjected to comprehensive phytochemical screening to determine the presence of key secondary metabolites, such as alkaloids, flavonoids, phenols, tannins, saponins, and terpenoids, utilizing standardized qualitative assays [9]. Each metabolite was detected using its specific reagent or reaction, providing an initial profile of the bioactive constituents. For a more quantitative assessment, the total flavonoid and phenolic contents were measured using spectrophotometric methods.

For the flavonoid quantification, the aluminum chloride colorimetric assay was employed, with rutin used as a reference standard. The absorbance of the reaction mixture was recorded at 415 nm, and the flavonoid content was expressed as micrograms of rutin equivalents per milliliter (µg RE/mL). Similarly, the total phenolic content was determined by the Folin–Ciocalteu reagent assay, with gallic acid serving as the standard. The absorbance of the phenolic reaction product was measured at 765 nm, and the phenolic content was reported as micrograms of gallic acid equivalents per milliliter (µg GAE/mL) [10]. These quantitative analyses provided essential insights into the phytochemical richness of the extracts and established a basis for linking specific bioactive components to their pharmacological effects.

### 2.3 Antioxidant activity evaluation

The antioxidant potential of the *A. tuberculata* extracts was determined through the 2,2-diphenyl-1-picrylhydrazyl (DPPH) free radical scavenging assay, a widely recognized method for assessing the capacity of compounds to neutralize free radicals [11]. In this assay, the DPPH radical, which exhibits a deep purple color, undergoes a color change to yellow upon reduction by an antioxidant. The extent of this color change reflects the scavenging activity of the sample.

Different concentrations of the crude extracts (ranging from 10 to 500 µg/mL) were prepared in methanol. A 2 mL aliquot of each concentration was mixed with 2 mL of freshly prepared DPPH solution (0.1 mM in methanol), and the reaction mixtures were incubated in the dark for 30 min to prevent the interference of light on the reaction kinetics. After incubation, the absorbance of each sample was measured at 517 nm using a UV–visible spectrophotometer. The percentage of DPPH radical inhibition was calculated using the following equation:

(1) \[ \% \text{DPPH radical}-\text{scavenging}=\hspace{.25em}(\text{Absorbance of control}-\text{Absorbance of test sample})\hspace{.25em}\times 100.]\]

The antioxidant capacity of the extracts was expressed as the IC50 value (µg/mL), defined as the concentration of the extract required to inhibit 50% of DPPH radicals. Lower IC50 values indicate higher antioxidant activity. Ascorbic acid was used as a positive control to benchmark the antioxidant efficacy of the plant extracts. The IC50 values were calculated from dose-response curves using nonlinear regression analysis, providing a quantitative measure of the antioxidant potential of the extracts. This antioxidant assay, in combination with the phytochemical analyses, allowed for a detailed evaluation of the relationship between the chemical composition of the *A. tuberculata* extracts and their bioactive properties, establishing a foundation for further pharmacological investigations in animal models.

### 2.4 Animal studies

Male Swiss albino mice, weighing between 20 and 25 g, were procured from the National Institutes of Health, Islamabad, Pakistan, for the *in vivo* pharmacological evaluation of *A. tuberculata* extracts. A total of 45 mice were acclimatized to laboratory conditions for 1 week prior to the experiments. The animals were housed in polypropylene cages and maintained under controlled environmental conditions: a temperature of 22 ± 2°C, relative humidity of 50 ± 5%, and a 12-h light/dark cycle. They were provided ad libitum access to a standard pellet diet and water throughout the study period.

### 2.5 Evaluation of analgesic activity

The analgesic potential of the EEAT was assessed using the acetic acid-induced writhing test, a well-established method for evaluating peripheral analgesic effects in rodents [12]. Mice were randomly divided into groups (*n* = 5 per group) and orally administered varying doses of the extract (100, 200, and 400 mg/kg body weight) or a reference drug, aspirin (150 mg/kg). After 30 min, each mouse received an intraperitoneal injection of 0.6% acetic acid (10 mL/kg) to induce a nociceptive response, characterized by abdominal writhing. The number of writhing episodes was recorded for each mouse over a 5 min observation period, starting 5 min after acetic acid administration. The percentage inhibition of writhing was calculated for each treatment group using the following equation:

(2) \[\hspace{8em}{\mathrm{Inhibition}}(\left \% )=\frac{({\mathrm{Number\; of\; writhing\; in\; control}}-{\mathrm{Number\; of\; writhing\; in\; test}})}{{\mathrm{Number\; of\; writing\; in\; control}}}\times 100.]\]

### 2.6 Evaluation of anti-inflammatory activity

The anti-inflammatory properties of the ethanolic extract were evaluated using the carrageenan-induced paw edema model, a widely used method for assessing acute inflammation in animal models [13]. Mice were divided into groups and treated orally with the extract (100, 200, and 400 mg/kg) or a reference drug, diclofenac sodium (10 mg/kg). One hour post-administration, acute inflammation was induced by injecting 0.1 mL of 1% carrageenan into the subplantar region of the right hind paw. Paw edema was measured at baseline (before carrageenan injection) and at 1, 2, 3, and 4 h post-injection using a plethysmometer. The degree of inflammation was determined by comparing the paw volume in treated mice to that of the control group, and the percentage inhibition of edema was calculated using the following equation:

(3) \[(\left \% ){\mathrm{inhibition}}{-}\frac{{V}_{{\mathrm{c}}}-{V}_{{\mathrm{t}}}}{{V}_{{\mathrm{c}}}}\times 100,]\]

where *V*_c_ is the volume of the paw in the control group (without treatment) and *V*_t_ is the volume of the paw in the treatment group (with extract or drug). This model provided insights into the anti-inflammatory potential of the extracts.

### 2.7 Evaluation of antipyretic activity

The antipyretic effect of the *A. tuberculata* ethanolic extract was evaluated using the yeast-induced pyrexia model, which mimics fever conditions in animals [14]. Pyrexia was induced by subcutaneous injection of Brewer’s yeast suspension (10 mL/kg of 20% yeast in saline) into the dorsum of the mice. After 18 h of yeast injection, rectal temperatures were measured using a digital thermometer, establishing a baseline of fever induction (B). The mice were then divided into groups and treated orally with the extract (100, 200, and 400 mg/kg) or a standard antipyretic drug, paracetamol (150 mg/kg). Rectal temperatures were recorded at 1, 2, 3, 4, and 5 h post-treatment. The percentage reduction in temperature was calculated using equation (4).

(4) \[{\mathrm{Percent\; reduction}}=\frac{B-{C}_{{\mathrm{n}}}}{B-A}\times 100,]\]

where *B* is temperature after pyrexia induction, *C*_n_ is temperature after 1, 2, 3, 4, and 5 h, and *A* is normal body temperature.

### 2.8 Evaluation of gastrointestinal effects

The gastrointestinal effects of the ethanolic extract were assessed using the charcoal meal transit test, which evaluates intestinal motility [15]. Mice were fasted for 18 h before the experiment and randomly assigned to receive either the extract (100, 200, and 400 mg/kg), atropine sulfate (5 mg/kg) as a positive control, or saline (10 mL/kg) as a negative control. Thirty minutes after the treatment, each mouse was administered an oral charcoal meal (10% charcoal suspension in 5% gum acacia, 0.5 mL). After 30 min, the mice were euthanized, and the distance traveled by the charcoal meal through the small intestine was measured and expressed as a percentage of the total length of the small intestine. Intestinal transit was calculated using the following equation:

(5) \[{\mathrm{Intestinal}}{\mathrm{t}}{\mathrm{ransit}}(\left \% )=\frac{D}{L}\times 100,]\]

where DDD is the distance traveled by the charcoal meal and LLL is the total length of the small intestine. This test provided insights into the prokinetic or inhibitory effects of the extract on gastrointestinal motility.

### 2.9 Statistical analysis

All experimental data were expressed as the mean ± standard error of the mean (SEM) and statistical significance was determined using one-way analysis of variance (ANOVA) [16], followed by Tukey’s post hoc test for multiple comparisons between groups. A *p*-value of less than 0.05 (*p* < 0.05) was considered statistically significant. All statistical analyses were performed using GraphPad Prism software (version X), ensuring rigorous and reliable interpretation of the results. The use of SEM and appropriate statistical tests ensured the precision and reliability of the observed pharmacological effects.


**Ethical approval:** All experimental protocols were conducted in strict accordance with the ethical guidelines for animal experimentation, as outlined by the Institutional Animal Ethics Committee, and followed the regulations of the Committee for the Purpose of Control and Supervision of Experiments on Animals. Ethical approval was obtained for all animal studies before their commencement, and every effort was made to minimize animal suffering and the number of animals used.

## 3 Results

The data presented herein elucidate the effects of the EEAT and other compounds on various physiological parameters in murine models. The findings demonstrate significant variations in extraction efficiencies of bioactive compounds, antioxidant activity, analgesic effects, anti-inflammatory properties, and temperature regulation. Collectively, these results highlight the potential medicinal value of EEAT and underscore its dose-dependent effects on physiological processes. Further exploration is warranted to comprehensively elucidate the therapeutic potential and underlying mechanisms of action of these compounds, thereby paving the way for potential applications in the management of diverse health conditions.

### 3.1 Quantitative analysis of flavonoid and phenol

**Figure 1:**
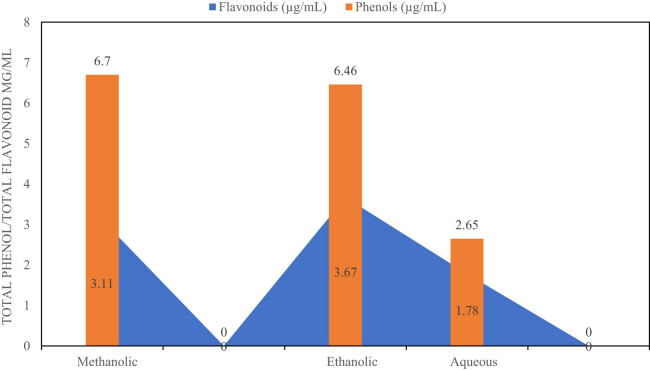
Quantitative analysis: flavonoid and phenol in *A. tuberculata* extracts.

Figure 1 presents a quantitative analysis of flavonoid and phenolic content across different extraction methods of *A. tuberculata*. Three extraction techniques were compared: methanolic, ethanolic, and aqueous. Notably, the ethanolic extract exhibited the highest mean values for both flavonoids (3.67 µg/mL) and phenols (6.46 µg/mL), indicating superior efficacy in extracting these bioactive compounds relative to the other methods. In contrast, the methanolic and aqueous extracts yielded lower mean values for flavonoids (3.11 and 1.78 µg/mL, respectively) and phenols (6.7 and 2.65 µg/mL, respectively). These results emphasize the significance of the ethanolic extraction method for obtaining bioactive compounds from *A. tuberculata*, warranting further investigation into its medicinal or nutritional implications and optimization of extraction techniques.

### 3.2 Antioxidant activity

**Figure 2:**
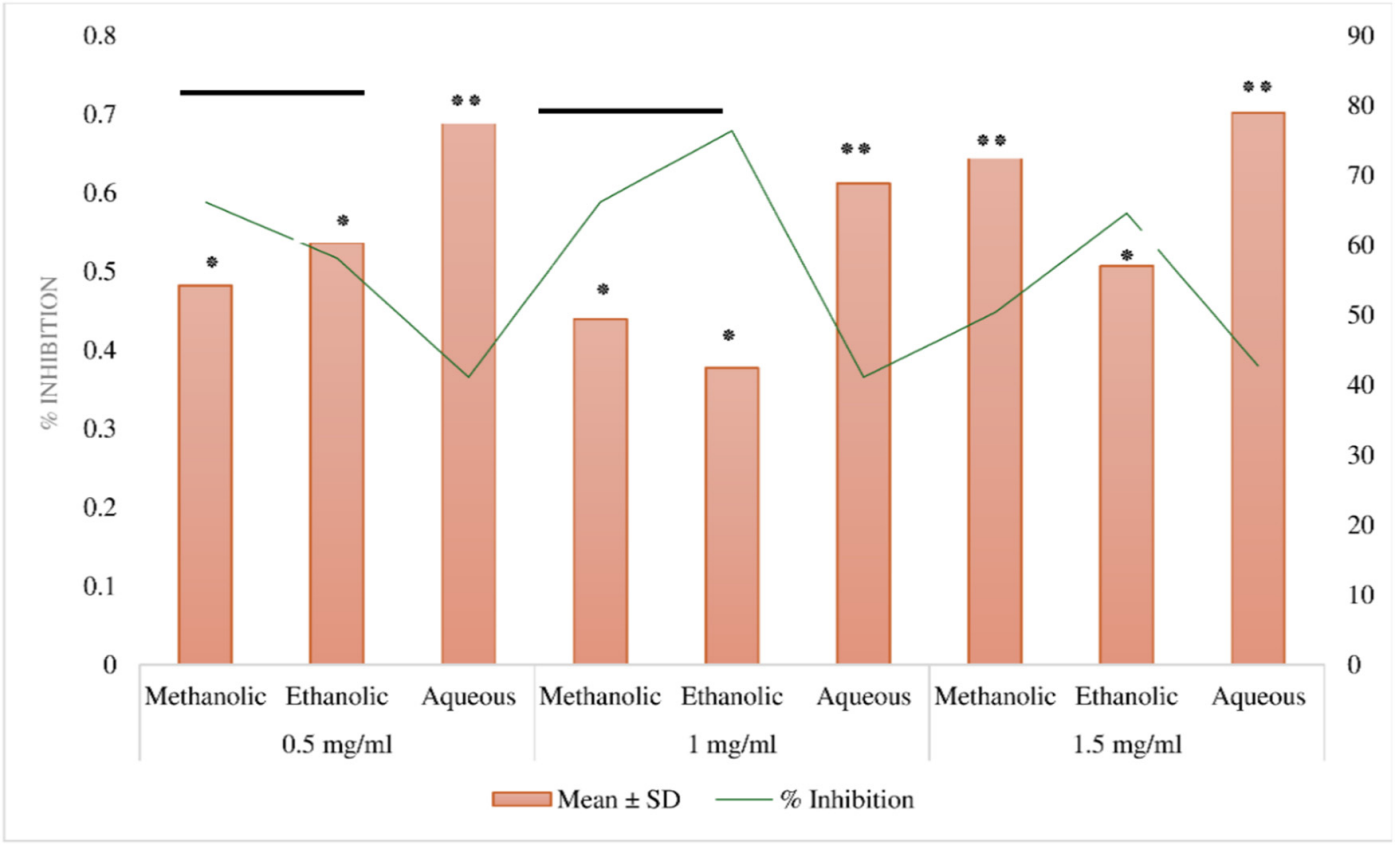
Antioxidant activity of *A. tuberculata* extracts at varying concentrations. *p* < 0.05 (*) a significant difference between the ethanolic and methanolic extracts. *p* < 0.01 (**) a highly significant difference between the ethanolic and aqueous extracts.

Figure 2 outlines the antioxidant activity of various extracts obtained from *A. tuberculata*, measured at different concentrations. The extracts were derived using methanolic, ethanolic, and aqueous solvents. Notably, at a concentration of 1 mg/mL, the ethanolic extract exhibited the highest mean antioxidant activity, with a value of 0.377 ± 0.011, and a significant inhibition percentage of 76.4% (*p* < 0.01). Conversely, the methanolic and aqueous extracts at the same concentration displayed lower mean antioxidant activity values of 0.439 ± 0.093 (*p* < 0.05) and 0.612 ± 0.055, respectively, alongside inhibition percentages of 66.2% (*p* < 0.05) and 41.1% (not significant). Similar trends were observed at other concentrations, with the ethanolic extract consistently demonstrating superior antioxidant activity compared to the methanolic and aqueous extracts (all *p* < 0.05). These findings underscore the robust antioxidant potential of the ethanolic extraction method in isolating bioactive compounds from *A. tuberculata*.

### 3.3 Analgesic activity

**Figure 3:**
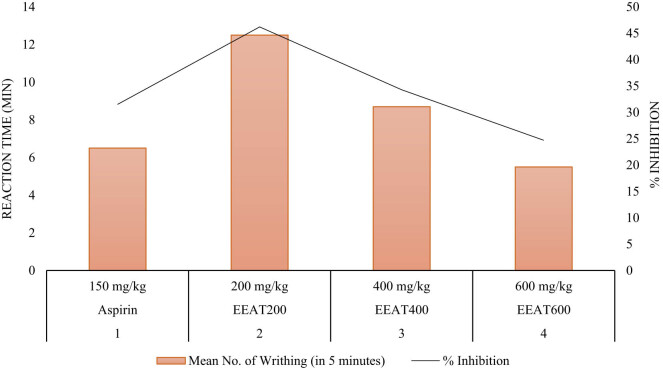
*A. tuberculata* extract’s analgesic activity in mice.

The analgesic activity of the EEAT was evaluated using the writhing response test, as detailed in Table 1 and Figure 3. Mice were allocated into five groups and administered different treatments, including aspirin as a standard reference. The “Mean Number of Writhing” values reflect the average number of writhing responses observed within a 5-min interval, while “% Inhibition” represents the percentage of writhing response inhibition relative to the control group. At a dosage of 200 mg/kg (EEAT200), the ethanolic extract elicited a significant reduction in the writhing response (12.5 ± 0.40) compared to the control group, indicating a pronounced analgesic effect (*p* < 0.01). In contrast, the control group and lower doses of the extract did not demonstrate significant reductions in writhing response. These findings suggest a dose-dependent analgesic effect of the ethanolic extract, with the 200 mg/kg dosage yielding the most promising results.

**Table 1:** Analgesic activity of EEAT in mice

Group	Treatment design	Dose	Mean no. of writhing (in 5 min)	% inhibition
1	N/S	10 mL/kg	25.5 ± 0.55	—
2	Aspirin	150 mg/kg	6.5 ± 0.35	31.50*
3	EEAT200	200 mg/kg	12.5 ± 0.40	46.20**
4	EEAT400	400 mg/kg	8.7 ± 0.60	34.28*
5	EEAT600	600 mg/kg	5.5 ± 0.30	24.70

A single asterisk (*) indicates that the difference observed is statistically significant at the *p* < 0.05 level, meaning there is less than a 5% probability that the result is due to chance. A double asterisk (**) denotes a higher level of statistical significance at the *p* < 0.01 level, suggesting an even lower probability (less than 1%) that the observed effect is random.

### 3.4 Anti-inflammatory activity

**Figure 4:**
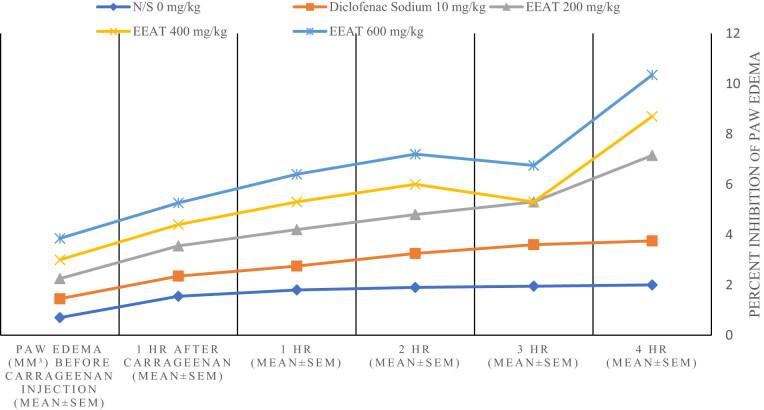
Comparing diclofenac sodium and *A. tuberculata* on mouse paw edema.

As shown in Table 2 and Figure 4, the control group (N/S) exhibited a baseline paw edema volume of 0.70 mm³ prior to carrageenan injection, which progressively increased to 2.00 mm³ at the 4-h mark. Treatment with Diclofenac sodium at a dose of 10 mg/kg resulted in a marked reduction in paw edema volume compared to the control group, with measurements ranging from 0.75 to 1.75 mm³ over the same period, suggesting a significant anti-inflammatory effect.

**Table 2:** Effects of diclofenac sodium and EEAT on carrageenan-induced hind paw edema in mice

Groups	Drug	Dose (mg/kg)	Paw edema (mm³) before carrageenan injection (mean ± SEM)	1 h after carrageenan (mean ± SEM)	1 h (mean ± SEM)	2 h (mean ± SEM)	3 h (mean ± SEM)	4 h (mean ± SEM)
1	N/S	—	0.70 ± 0.04	1.55 ± 0.03	1.80 ± 0.03	1.90 ± 0.03	1.95 ± 0.05	2.00 ± 0.03
2	Diclofenac sodium	10	0.75 ± 0.05	0.80 ± 0.03	0.95 ± 0.03	1.35 ± 0.03	1.65 ± 0.03	1.75 ± 0.05*
3	EEAT	200	0.80 ± 0.03	1.20 ± 0.05	1.45 ± 0.03	1.55 ± 0.05	1.70 ± 0.05	3.40 ± 0.03**
4	EEAT	400	0.75 ± 0.03	0.85 ± 0.03	1.10 ± 0.06	1.20 ± 0.03	1.30 ± 0.03	1.55 ± 0.03
5	EEAT	600	0.85 ± 0.04	0.86 ± 0.05	1.10 ± 0.03	1.20 ± 0.03	1.45 ± 0.03	1.65 ± 0.03

*p* < 0.05 is significant compared with standard drug.

EEAT = ethanolic extract of *A. tuberculata*; N/S = normal saline.

A single asterisk (*) indicates that the difference observed is statistically significant at the *p* < 0.05 level, meaning there is less than a 5% probability that the result is due to chance. A double asterisk (**) denotes a higher level of statistical significance at the *p* < 0.01 level, suggesting an even lower probability (less than 1%) that the observed effect is random.

The EEAT displayed variable effects contingent on the administered dosage. At 200 mg/kg, EEAT demonstrated a similar trend to the control group initially but exhibited a notable increase in paw edema volume at the 4-h time point, with an even more pronounced increase observed at a dosage of 400 mg/kg. Conversely, at a dosage of 600 mg/kg, EEAT resulted in a slight reduction in paw edema volume relative to the control group, suggesting a potential dose-dependent effect. These findings highlight the complex nature of the anti-inflammatory effects of the tested treatments and underscore the necessity for further investigations to elucidate the optimal dosages and therapeutic potential of the ethanolic extract in managing inflammatory conditions.

### 3.5 Antipyretic activity

**Figure 5:**
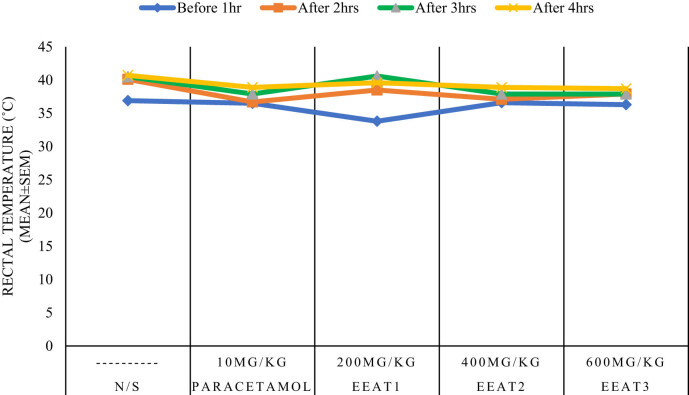
Ethanol extract and paracetamol on fever from brewer’s yeast in mice.

Table 3 and Figure 5 present data concerning the effects of various drugs on body temperature regulation over a specified timeframe. The non-specific drug (N/S) exhibited a baseline temperature of approximately 36.9°C, with a notable increase to 40.1°C after 2 h, indicating its potential influence on body temperature modulation. Paracetamol, administered at 10 mg/kg, produced a milder effect compared to the non-specific drug, with an initial temperature of 36.5°C, gradually increasing to 36.7°C after 2 h and displaying further increments thereafter.

**Table 3:** Impact of EEAT and paracetamol on fever induced by Brewer’s yeast in mice

Drug design	Treatment (mg/kg)	Before 1 h	After 2 h	After 3 h	After 4 h
N/S	—	36.9 ± 0.04	40.1 ± 0.04	40.5 ± 0.04	40.7 ± 0.04
Paracetamol	10	36.5 ± 0.04	36.7 ± 0.02	37.9 ± 0.02	38.9 ± 0.02
EEAT1	200	33.8 ± 2.00	38.5 ± 0.03	40.6 ± 0.04	39.6 ± 0.04
EEAT2	400	36.6 ± 0.02	37.1 ± 0.02	37.9 ± 0.04	38.9 ± 0.04
EEAT3	600	36.3 ± 0.06	37.9 ± 0.05	37.9 ± 0.05	38.7 ± 0.05

*p* < 0.05 is significant compared with the standard drugs key.

EEAT = ethanolic extract of *Apteranthes tuberculate*; N/S = normal saline.

In contrast, EEAT1 (200 mg/kg) displayed a distinctive pattern, with an initial temperature drop to 33.8°C followed by a steady rise to 40.6°C at the 3 h mark. Doses EEAT2 (400 mg/kg) and EEAT3 (600 mg/kg) exhibited similar dose-dependent responses, with temperature changes reflecting moderate increases over time. Statistical analyses, including *t*-tests or ANOVA, are warranted to ascertain the significance of these temperature changes while understanding the clinical relevance is essential for evaluating the efficacy and safety of these pharmacological interventions. Furthermore, the observed dose–response relationship highlights the importance of dosage adjustment for optimizing therapeutic outcomes while minimizing adverse effects. In conclusion, these data offer valuable insights into the pharmacological effects of these agents on body temperature regulation, warranting further investigation through clinical trials to elucidate their clinical implications and refine therapeutic applications.

### 3.6 Gastrointestinal effects

**Figure 6:**
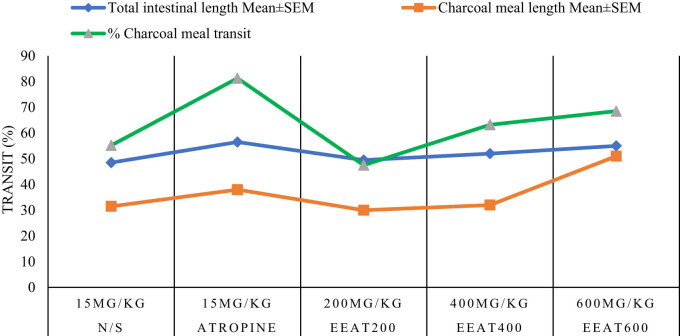
Ethanol extract and deactivated charcoal on mouse gastric spasms.

Table 4 and Figure 6 investigate the impact of the EEAT on deactivated charcoal-induced gastric spasms in mice. Each experimental group received specific treatments, and parameters such as total intestinal length, charcoal meal length, and the percentage of charcoal meal transit were meticulously measured. Notably, both EEAT 400 (400 mg/kg) and EEAT 600 (600 mg/kg) exhibited significant increases in total intestinal length and charcoal meal length compared to the control, while improvements in charcoal meal transit percentages were less pronounced. EEAT 200 (200 mg/kg) displayed comparable intestinal length but exhibited slightly lower efficacy in enhancing charcoal meal transit.

**Table 4:** Effect of EEAT and deactivated charcoal-induced gastric spasms in mice

Drug	Treatment design (mg/kg)	Total intestinal length mean ± SEM	Charcoal meal length mean ± SEM	% charcoal meal transit
N/S	15	48.5 ± 0.35	31.5 ± 0.22	55.2
Atropine	15	56.5 ± 0.35	38.0 ± 0.50	81.3
EEAT200	200	49.5 ± 0.28	30.0 ± 0.18	47.5
EEAT400	400	52.0 ± 0.48	32.0 ± 0.30	63.2
EEAT600	600	55.0 ± 0.21	51.0 ± 0.12	68.5

*p* < 0.05 is significant compared with the standard drug.

Statistical analyses essential for assessing the reliability of these results were not provided in the table. Therefore, obtaining significance values through appropriate statistical tests, such as *t*-tests or ANOVA, is critical to further validate the observed effects. Overall, these findings provide significant insights into the potential therapeutic effects of EEAT on gastric spasms in mice, suggesting avenues for further research aimed at optimizing dosage and understanding underlying mechanisms to improve treatment outcomes in gastrointestinal disorders.

## 4 Discussion

The findings of this study provide significant insights into the comparative efficacy of different extraction methods employed for isolating bioactive compounds from *A. tuberculata*, with methanolic, ethanolic, and aqueous extractions being the primary focus. Our results reveal distinct variations in the yields of flavonoids and phenolic compounds, as well as differences in antioxidant activity across these extraction methods. These observations are consistent with prior research, underscoring the pivotal role of extraction solvents in maximizing the retrieval of bioactive phytochemicals.

Specifically, our results corroborate earlier findings that ethanol is a highly effective solvent for extracting bioactive compounds such as flavonoids and phenols. Studies have shown that ethanol, due to its polar and non-polar properties, can solubilize a wider spectrum of phytochemicals compared to methanol or water [17]. Our data similarly highlight the superior efficacy of ethanolic extraction in *A. tuberculata*, where higher concentrations of phenolic and flavonoid compounds were obtained. This supports ethanol’s established role in optimizing phytochemical extraction from various botanical sources, particularly within the same botanical family as *A. tuberculata*.

The antioxidant activity of the ethanolic extract aligns with previous investigations that demonstrate the superior antioxidant potential of ethanol-based extracts over methanolic and aqueous extracts. Research by Peralta et al. [18] shows that ethanol extracts exhibit higher antioxidant activity, attributable to the higher presence of polyphenols and flavonoids, which are key contributors to antioxidant properties. In our study, the EEAT exhibited the highest antioxidant capacity, which can be attributed to the concentration of these bioactive compounds, further emphasizing the importance of solvent choice in maximizing pharmacological efficacy.

Moreover, the comparative analysis of the analgesic, anti-inflammatory, antipyretic, and gastrointestinal effects of the EEAT in animal models further elucidates its multifaceted pharmacological potential. The analgesic activity, as illustrated in Table 1, shows that EEAT, particularly at 200 mg/kg, significantly reduces the acetic acid-induced writhing response in mice, suggesting a potent dose-dependent analgesic effect. These findings are consistent with studies on other medicinal plants, where plant-derived extracts exhibit similar analgesic properties at comparable doses [19,20,21]. However, further investigation into the precise molecular mechanisms underlying the analgesic effects of EEAT is warranted to better understand its therapeutic applications in pain management.

The anti-inflammatory properties of EEAT, assessed through the carrageenan-induced paw edema model (Table 2), reveal a complex dose–response relationship. While the 200 mg/kg dose demonstrated significant anti-inflammatory activity, higher doses exhibited varied responses, suggesting the presence of multiple pathways or modulatory mechanisms influencing inflammation. Previous studies [22,23] have also noted similar dose-dependent variations in anti-inflammatory efficacy in plant extracts, highlighting the need for further research to optimize dosing and elucidate the underlying mechanisms of action.

The antipyretic activity of EEAT, as depicted in Table 3, shows a distinctive pattern of temperature modulation in mice, where a 200 mg/kg dose elicited a marked initial decrease in body temperature, followed by a gradual rise. This biphasic temperature response indicates a complex interplay between thermoregulatory pathways and phytochemicals present in the extract, necessitating further research to unravel the mechanisms involved and their potential implications in treating pyrexia [24,25]. The dose-dependent effects on body temperature observed in higher doses further emphasize the importance of determining the optimal therapeutic dose for fever management.

In terms of gastrointestinal effects, Table 4 presents intriguing data on the impact of EEAT on charcoal meal transit in mice. The extract at 400 and 600 mg/kg demonstrated notable improvements in intestinal motility, as indicated by the increased transit distance of the charcoal meal. However, the variation in efficacy across doses suggests that the pharmacological effects of EEAT on gastrointestinal motility may depend on specific dose thresholds or combinations of bioactive compounds. These findings echo similar observations in the literature, where plant extracts have shown promise in regulating gastrointestinal functions, though the mechanisms require further investigation [26].

This comprehensive assessment of *A. tuberculata* extracts offers valuable insights into the phytochemical composition and pharmacological potential of the plant, particularly through ethanolic extraction. The multifaceted pharmacological activities observed in this study, including analgesic, anti-inflammatory, antipyretic, and gastrointestinal regulatory effects, demonstrate the therapeutic potential of *A. tuberculata* in various health conditions. Nonetheless, further in-depth studies are essential to elucidate the underlying mechanisms, optimize dosing regimens, and validate these findings through *in vivo* efficacy studies. This research lays the foundation for future explorations into the potential applications of *A. tuberculata* extracts in the development of novel therapeutic agents.

## 5 Conclusion

This study provides a comprehensive assessment of *A. tuberculata* extracts, highlighting their medicinal potential and underscoring the critical role of extraction methods in optimizing the bioactivity of herbal compounds. By comparing methanolic, ethanolic, and aqueous extraction techniques, we observed significant variations in the yield of bioactive compounds such as flavonoids and phenols, as well as differences in antioxidant activity. The EEAT emerged as the most potent, exhibiting superior antioxidant properties and a promising pharmacological profile *in vivo*. Specifically, EEAT demonstrated notable analgesic, anti-inflammatory, antipyretic, and gastrointestinal regulatory effects in animal models, suggesting its therapeutic potential. Despite these promising findings, further research is essential to fully elucidate the mechanisms underlying these pharmacological effects and to optimize dosing regimens for clinical applications. The dose-dependent variations observed across different assays highlight the importance of identifying the precise bioactive constituents responsible for these effects, as well as the need for a deeper understanding of their interactions with biological systems. Additionally, while *in vitro* and *in vivo* results are encouraging, translating these findings into practical therapeutic interventions will require more extensive preclinical and clinical studies. This study contributes to the growing body of knowledge on *A. tuberculata* by providing detailed phytochemical and pharmacological evaluations. The multifaceted bioactivity of EEAT positions it as a promising candidate for further development in the treatment of various health conditions. Continued research in this area will not only expand our understanding of *A. tuberculata*’s therapeutic potential but also pave the way for the integration of its bioactive compounds into evidence-based medicinal applications, bridging the gap between traditional herbal medicine and modern healthcare advancements.

## Statement of originality

The authors declare that this manuscript is original, has not been published before, and is not currently being considered for publication elsewhere. The authors confirm that the manuscript has been read and approved by all named authors and that there are no other persons who satisfied the criteria for authorship but are not listed. The authors further confirm that the order of authors listed in the manuscript has been approved by all of us. The authors understand that the corresponding author is the sole contact for the editorial process. The corresponding author is responsible for communicating with the other authors about progress, submissions of revisions, and final approval of proofs.
